# Trends and outcomes in colorectal cancer surgery: a multicenter cross-sectional study of minimally invasive versus open techniques in Germany

**DOI:** 10.1007/s00464-024-11210-1

**Published:** 2024-08-29

**Authors:** Andreas Krieg, Ernst W. Kolbe, Michael Kaspari, Sarah Krieg, Sven H. Loosen, Christoph Roderburg, Karel Kostev

**Affiliations:** 1 Department of General and Visceral Surgery, Thoracic Surgery and Proctology, Medical Campus OWL, University Hospital Herford, Ruhr University Bochum, Schwarzenmoorstr. 70, 32049 Herford, Germany; 2 Department of Inclusive Medicine, University Hospital Ostwestfalen-Lippe, Bielefeld University, 33617 Bielefeld, Germany; 3 Department of Gastroenterology, Hepatology and Infectious Diseases, Medical Faculty of Heinrich Heine University Duesseldorf, University Hospital Duesseldorf, 40225 Duesseldorf, Germany; 4 Epidemiology, IQVIA, 60549 Frankfurt, Germany

**Keywords:** Colorectal cancer, Minimal invasive surgery, Laparoscopic surgery, Robot-assisted surgery, Colorectal surgery

## Abstract

**Background:**

The objective of this study was to assess the trend from open to modern minimally invasive (laparoscopic and robot-assisted) surgical techniques for colorectal cancer (CRC) in Germany, with a particular focus on hospital mortality, postoperative complications, and length of hospital stay.

**Methods:**

A multicenter cross-sectional study was conducted using data from 36 German hospitals, encompassing 1,250,029 cases from January 2019 to December 2023. The study included all hospitalized patients aged ≥ 18 with CRC who underwent surgery. Surgical cases were categorized as open or minimally invasive. Outcomes assessed included in-hospital mortality, morbidity, and hospital length of stay. Statistical analyses involved multivariable logistic and linear regression models adjusted for main diagnosis, metastasis presence, age, sex, and comorbidities.

**Results:**

The study included 4525 CRC cases: 2767 underwent open surgery and 1758 underwent minimally invasive surgery (173 robotic). In-hospital mortality was significantly higher in open surgery (6.1% vs. 1.7%). Open surgery was also significantly associated with higher rates of acute post-hemorrhagic anemia (OR: 2.38; 95% CI: 1.87–3.02), respiratory failure (OR: 1.71; 95% CI: 1.34–2.18), and intraoperative and postprocedural complications (OR: 3.64; 95% CI: 2.83–4.70). Average hospital stay was longer for open surgery (19.5 days vs. 11.0 days).

**Conclusion:**

Despite the advantages of minimally invasive surgery, including reduced mortality, morbidity, and shorter hospital stays, open surgery remains the predominant approach for CRC in Germany. These findings underscore the need for increased adoption of minimally invasive techniques and highlight the potential benefits of shifting toward minimally invasive methods to enhance the overall quality of CRC care.

**Graphical abstract:**

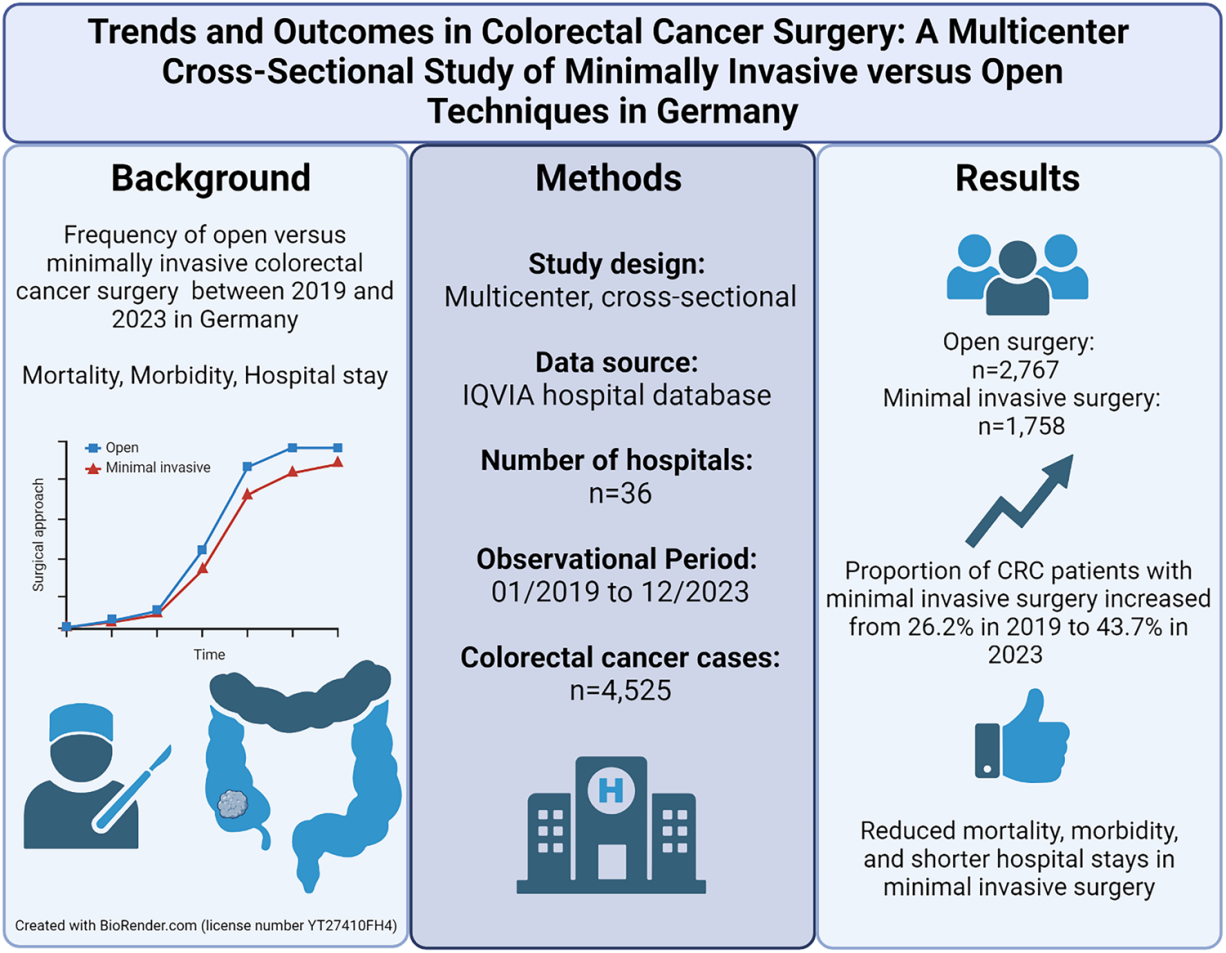

In Germany, approximately one in nine cancers affects the colon or rectum, with an age-standardized incidence in 2020 of 29.6/100,000 in women and 46.2/100,000 in men [1]. It is noteworthy that more than half of the cases occur in patients over the age of 70 [1]. This corresponds to a comparatively high mean age of onset of 75 years (women) and 71 years (men). The treatment of first choice with curative intent is surgical resection, which is accompanied by neoadjuvant therapy in rectal cancer depending on the extent of the primary tumor and/or involvement of the regional lymph nodes. Prior to laparoscopic colectomy’s introduction in 1991 [2, 3], laparotomy constituted the standard procedure. Nevertheless, the short-term advantages of laparoscopic colectomy over open colon resection, including shorter hospital stays, fewer surgical complications, improved early postoperative quality of life, earlier postoperative bowel movements, and less pain on the first postoperative day, have now been demonstrated [4].

Subsequently, the debate focused on possible differences in oncologic outcomes between the two techniques. A Cochrane review showed that laparoscopic resection of colorectal cancer (CRC) is associated with long-term outcomes that are not different from open surgery [5]. Theophilus and colleagues [6] included four multicenter randomized controlled trials (RCTs) in their 2014 meta-analysis comparing the open versus laparoscopic approach specifically for colon cancer: Clinical Outcomes of Surgical Therapy (COST) [7], Colon Cancer Laparoscopic or Open Resection (COLOR) [8], Conventional versus Laparoscopic-Assisted Surgery in Patients with Colorectal Cancer (CLASICC) [9], and the Australasian Laparoscopic Colon Cancer Study (ALCCaS) [10], as well as the Barcelona Trial, a monocentric RCT [11]. This meta-analysis impressively confirmed no difference in long-term survival between the two approaches.

With the introduction of robot-assisted laparoscopic surgery (RAS) following the approval of the da Vinci® Surgical System in 2000, minimally invasive surgery was also revolutionized in the field of colorectal surgery. The potential technical advantages of RAS over conventional laparoscopic or open surgery include stable, high-resolution 3D optics, multiple instruments with wrists that allow for better maneuverability, and digital processing to scale movements and filter out physiological tremor, which in turn ensures more precise tissue dissection. In addition, the ergonomic advantages that allow a neutral and comfortable posture and thus prevent mental and physical fatigue must be taken into account [12]. Nevertheless, the cost–benefit analysis of RAS continues to be a source of contention within the surgical community and among hospital managers [13–16].

In Germany, a registry study covering approximately 28% of the German population between 2002 and 2011 revealed that a conventional open approach was still the preferred choice for 89.3% of all colon cancers [17]. Consequently, laparoscopic colon surgery has not yet attained a significant degree of importance in Germany. Importantly, the extent to which the frequency of the different access routes has changed with the introduction of RAS has been little investigated to date. The objective of this study is therefore to evaluate the surgical approach to CRC on the basis of a multicenter study based on a hospital database from the last five years and to gain a better insight into surgery complications and mortality.

## Materials and methods

### Data source

This multicenter cross-sectional study was based on data from the hospital database (Company: IQVIA), which contained data from 36 hospitals and a total of 1,250,029 hospitalization cases between January 1, 2019 and December 31, 2023 across Germany, including specialized hospitals, primary care hospitals, maximum care, standard care, and university hospitals. The dataset describes a standardized data format that the hospitals transmit to the Reimbursement Institute for Hospitals (InEK) following §21 of the Hospital Compensation Act (KHEntgG). The individual treatment episodes included in §21 dataset of a case are grouped using special grouper software developed by 3 M Health Information Systems and IQVIA. In addition, the export files generated by the software are anonymized (e.g., case and patient number) for data protection reasons before transmission. Thus, patient data were assessed in aggregate form, with no personal data being available, and the authors did not have any access to identifiable records at any point in the analysis of the data. German law permits the use of electronic anonymized medical records for scientific studies under certain criteria. According to this legislation, it is not required to obtain informed consent from patients or approval from an Institutional Review Board (IRB) for this type of study using anonymous data. The study followed the strengthening the reporting of observational studies in epidemiology (STROBE) Statement guidelines for reporting cross-sectional studies.

### Study population

**Fig. 1:**
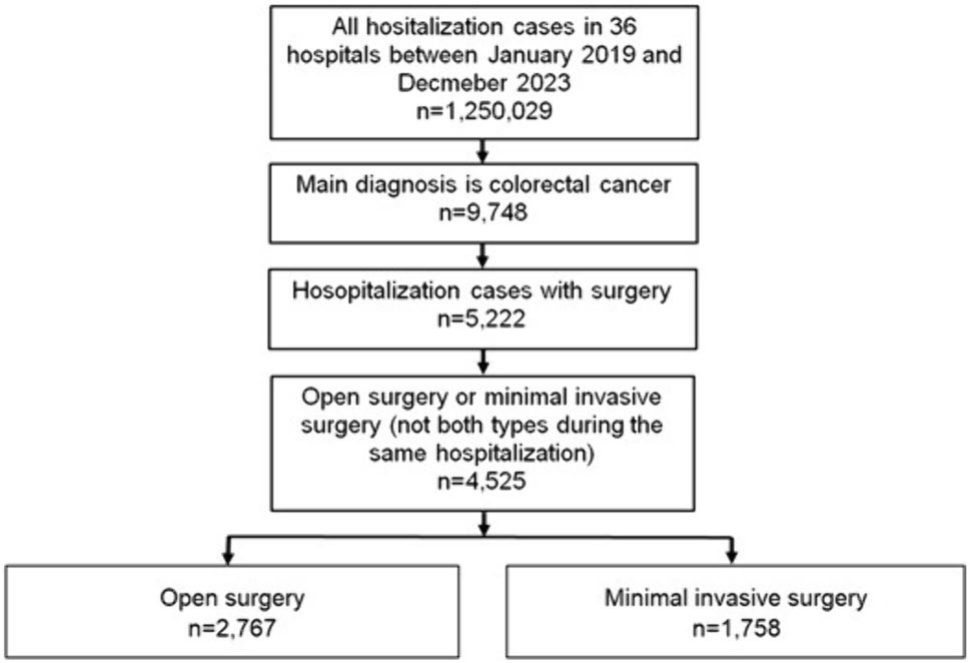
Selection of study patients

The cross-sectional study included all hospitalized patients who met the following inclusion criteria: (1) aged ≥ 18, (2) admissions for colorectal cancer (ICD-10: C18-C20) between January 2019 and December 2023, and (3) open or minimal invasive (laparoscopic or robotic) surgery. We have excluded patients without surgery or with both open and laparoscopic surgery during a hospital stay. All surgery cases were classified in open and minimal invasive (laparoscopic or robotic) surgery (Fig. 1).

### Study outcome

The outcome of the study was the prevalence of in-hospital mortality and complications, including acute post-hemorrhagic anemia (ICD-10: D62), intraoperative and postprocedural complications and disorders of digestive system (ICD-10: K91), complications of procedures, not elsewhere classified (ICD-10: T81), respiratory failure (ICD-10: J96), as well as length of hospital stay as a function of open surgery compared to minimal invasive surgery. The dataset contains death as one of the discharge types.

### Statistical analyses

Baseline characteristics of study patients contained age groups (≤ 50, 51–60, 61–70, 71–80, > 80 years), sex, main diagnosis including colon cancer (ICD-10: C18), rectal cancer (ICD-10: C20), rectosigmoid junction cancer (ICD-10-C19), presence of lymph node metastases (ICD-10: C77) and distant metastases (ICD-10: C78, C79), and co-diagnosis, including diabetes mellitus (ICD-10: E10-E14), lipid metabolism disorders (ICD-10: E78), hypertension (ICD-10: I10), coronary heart disease (ICD-10: I25), atrial fibrillation and flutter (ICD-10: I48), heart failure (ICD-10: I50), obesity (ICD-10: E66), paralytic ileus and intestinal obstruction (ICD-10: K56), and peritonitis (ICD-10: K65).

Differences in the sample characteristics and diagnosis prevalence between open surgery cases and minimal invasive surgery cases were compared using the Wilcoxon test for continuous variables, and Chi^2^ test for categorical variables.

To access the associations between open surgery and in-hospital mortality as well as complications, multivariable logistic regression analyses were conducted adjusted for main diagnosis, metastasis presence, age, sex, and comorbidities. The results of the logistic regression models were given as the odds ratio (OR) for open surgery compared to minimal invasive surgery. Finally, the associations between open surgery compared to minimal invasive surgery and hospital length of stay (dependent variable) were analyzed with multivariable linear regression models also adjusted for main diagnosis, metastasis presence, age, sex, and comorbidities. *P* values < 0.05 were considered statistically significant only. All analyses were done using SAS 9.4 (SAS Institute, Cary, US).

## Results

### Baseline characteristics

**Fig. 2:**
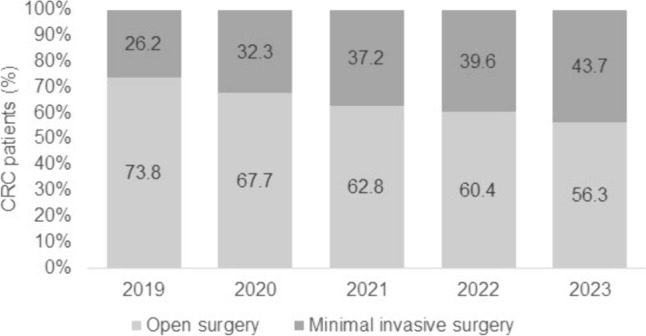
Proportion of CRC patients with open and minimal invasive surgery (%)

The present study included 2767 CRC cases with open surgery and 1758 CRC cases with minimal invasive (of them, only 173 robotic, 9.8%) surgery. Of the 2913 patients with colon cancer, 113 patients with rectosigmoid junction cancer, and 1499 patients with rectal cancer, only 34.7%, 53.1%, and 45.8%, respectively, underwent minimally invasive surgery. However, the proportion of CRC patients with minimal invasive surgery increased from 26.2% in 2019 to 43.7% in 2023 (Fig. 2).

Patients with open surgery were slightly older (70.8 vs. 68.7 years), with higher proportion of colon (68.7% vs. 57.5%) and lower proportion of rectal cancer (29.4% vs. 39.1%) as well as with higher proportion of distant metastases (21.0% vs. 14.1%). The prevalence of heart failure (8.8% vs. 4.7%), obesity (10.6% vs. 6.0%), paralytic ileus and intestinal obstruction (20.1% vs. 7.5%), and peritonitis (11.0% vs. 2.4%) was significantly higher in open surgery than in minimal invasive cases (Table 1).

**Table 1:** Baseline characteristics of the study sample

	Cases with open surgery (*n* = 2767)	Cases with minimal invasive surgery (*n* = 1758)	*p* value
Mean age			
Mean age (SD)	70.8 (12.2)	68.7 (12.5)	< 0.001
< 60 years, n (%)	563 (20.3)	443 (25.2)	< 0.001
60–69 years, n (%)	702 (25.4)	460 (26.2)	
70–79 years, n (%)	804 (29.1)	518 (29.5)	
80 + years, n (%)	698 (25.2)	337 (19.1)	
Gender, n (%)			
Female	1278 (46.2)	461 (43.3)	0.056
Male	1489 (53.8)	997 (56.7)	
Main diagnosis, n (%)			
Colon cancer	1902 (68.7)	1011 (57.5)	< 0.001
Rectal cancer	812 (29.4)	687 (39.1)	
Rectosigmoid junction cancer	53 (1.9)	60 (3.4)	
Secondary diagnosis, n (%)			
Lymph node metastases	702 (25.4)	299 (17.0)	< 0.001
Distant metastases	581 (21.0)	253 (14.4)	< 0.001
Diabetes mellitus	554 (20.0)	300 (17.1)	0.013
Lipid metabolism disorders	344 (12.4)	259 (14.7)	0.027
Hypertension	1439 (52.0)	866 (49.3)	0.072
Coronary heart disease	282 (10.2)	151 (8,6)	0.074
Atrial fibrillation and flutter	386 (14.0)	212 (12.1)	0.067
Heart failure	243 (8.8)	82 (4.7)	< 0.001
Obesity	295 (10.6)	106 (6.0)	< 0.001
Paralytic ileus and intestinal obstruction	556 (20.1)	131 (7.5)	< 0.001
Peritonitis	305 (11.0)	42 (2.4)	< 0.001

### Prevalence of in-hospital mortality and complications

Overall, in-hospital mortality (6.1% vs. 1.7%), acute post-hemorrhagic anemia (16.1% vs. 5.6%), respiratory failure (13.1% vs 5.9%), intraoperative and postprocedural complications and disorders of digestive system (17.7% vs. 4.6%), and complications of procedures, not elsewhere classified(17.8% vs. 4.6%) were higher in open surgery cases compared to minimal invasive surgery cases. Based on a multivariable regression model, open surgery was significantly associated with higher in-hospital mortality (OR: 1.97; 95% CI: 1.29–3.03), acute post-hemorrhagic anemia (OR: 2.38; 95% CI: 1.87–3.02), respiratory failure (OR: 1.71; 95% CI: 1.34–2.18), intraoperative and postprocedural complications and disorders of digestive system (OR: 3.64; 95% CI: 2.83–4.70), and complications of procedures, not elsewhere classified (OR: 3.86; 95% CI: 3.00–4.97) (Table 2).

**Table 2:** Association of open surgery as compared to minimal invasive surgery and in-hospital mortality and complications (multivariable logistic regression)

	Prevalence among patients with open surgery (N, %)	Prevalence among patients with minimal invasive surgery (N, %)	OR (95% CI)	*P* value
In-hospital mortality	168 (6.1)	29 (1.7)	1.97 (1.29–3.03)	0.002
Acute post-hemorrhagic anemia	444 (16.1)	99 (5.6)	2.38 (1.87–3.02)	< 0.001
Respiratory failure	363 (13.1)	104 (5.9)	1.71 (1.34–2.18)	< 0.001
Intraoperative and postprocedural complications and disorders of digestive system	490 (17.7)	81 (4.6)	3.64 (2.83–4.70)	< 0.001
Complications of procedures, not elsewhere classified	492 (17.8)	80 (4.6)	3.86 (3.00–4.97)	< 0.001

### Hospital length of stay

Table 3 shows the results of multivariable linear regression analysis. The length of hospital stay was higher in open surgery cases (19.5 days) compared to minimal invasive surgery cases (11.0 days). Open surgery was significantly associated with + 6.3 days longer hospital stay (*p* < 0.001). This association was confirmed for colon and rectal cancer patients separately (Table 3).

**Table 3:** Association of open surgery as compared to minimal invasive surgery and length of hospital stay (multivariable linear regression regression)

Main diagnosis	Length of hospital stay among patients with open surgery (days; mean, SD)	Length of hospital stay among patients with minimal invasive surgery (days; mean, SD)	Difference in days (ß coefficient)	*P* value
Total	19.5 (14.8)	11.0 (9.2)	+ 6.3	< 0.001
Colon cancer	19.6 (14.2)	10.4 (8.8)	+ 6.4	< 0.001
Rectal cancer	19.1 (16.4)	11.8 (9.2)	+ 6.0	< 0.001

## Discussion

In our multicenter cross-sectional study, a total of 4525 patients from 36 German hospitals who underwent surgery for CRC between 2019 and 2023 were included. The results indicate that despite the development of minimally invasive surgery, the majority of CRC patients in Germany are still surgically treated via an open approach (56.3%). Of note, only 9.8% of minimally invasive surgeries were performed using RAS. While cancers of the rectosigmoid junction and rectum were most commonly treated with minimally invasive surgery, only 34.7% of patients with colon cancer underwent minimally invasive surgery. Although the data are from a different database, a comparison with earlier publications by Benz et al. [17] and Völkel et al. [18], which reported a laparoscopic proportion of 10.7% for the period 2002 to 2011 and 16% in 2013, respectively, shows that the development of minimally invasive oncologic colorectal surgery has increased more slowly than in other countries. A recently published comparative study including data from the Dutch ColoRectal Audit (DCRA), the Swedish ColoRectal Cancer Registry (SCRCR), the National Bowel Cancer Audit (NBOCA) in England and Wales, and the Bowel Cancer Outcomes Registry (BCOR) from Australia and New Zealand reported that 55.7% of rectal cancer resections were still performed open between 2010 and 2019 [19]. In addition, the conversion rate was 12.3%. Of note, the most striking trend was seen in the Netherlands. Since 2015, more than 80% of procedures there have been performed laparoscopically. In contrast, the proportion of laparoscopic rectal resections was lower in Sweden exceeding the level of 60% in 2018. Comparable data were provided by a retrospective analysis using the American College of Surgeons National Surgical Quality Improvement Program (ACS-NSQIP) databases from 2012 to 2021, which showed that in 2012, colectomies were performed in equal proportions laparoscopically and conventionally open [20]. Interestingly, in this context, Ferrari et al. observed an increase in laparoscopic procedures in the third quarter of 2014 (58.2%), followed by a decrease to nearly 50% until 2021 [20]. Within the same time period, the proportion of robotic procedures increased from 0.0% to 18.0%, while the proportion of open procedures continuously decreased. Notably, the study also predicts that RAS will be the most commonly used surgical technique for colorectal surgery in the U.S.A. by 2024.

When interpreting our results, however, it must be taken into account that the proportion of patients with distant metastases, ileus, or peritonitis, possibly as a sign of colonic perforation, was 1.5–4.6 times higher in the group of patients who underwent conventional open surgery. Therefore, it is likely that our group includes patients who underwent primary open exploration for acute abdomen or synchronous abdominal metastasis. In addition, the laparotomy group had a significantly higher proportion of overweight patients. However, it should be emphasized that none of the previous studies included such factors in their analysis.

Despite these observations, the proportion of patients who underwent minimally invasive surgery is remarkably low when compared internationally. High-quality randomized controlled trials (RCTs) and meta-analyses have been available for years to dispel any doubts about the value of minimally invasive surgery for CRC. In addition, every hospital in Germany should have the necessary technical equipment for these procedures. Nevertheless, other causes must be considered. First, it is evident that an adequate infrastructure for the training and supervision of surgeons is essential to ensure safe performance. However, in Germany, laparoscopic education and training has not yet been integrated into specialist training. In this context, simulators or simulation models (i.e., animal model, cadaver model, 3D-printed models, Virtual reality [VR] simulation) can be considered helpful for further training [21]. Although the proportion of hospitals with simulators in Germany has increased since previous surveys, their use remains highly heterogeneous [22]. Second, the implementation of specialized training programs can lead to an increase in laparoscopic colorectal procedures at the respective institutions and also reduce morbidity and mortality [23]. Furthermore, it can be assumed that the institutional implementation of a proctoring program, as is known for RAS, has a positive influence on the increase in laparoscopic procedures in colon surgery [24]. On the other hand, a specialized section for CRC surgery is rather rare in Germany. The training program for abdominal surgeons in Germany remains relatively general in nature, conferring certification as either a general or visceral surgeon, with the option of pursuing additional certification in special visceral surgery. Consequently, many physicians in general and visceral surgery departments in Germany are highly experienced surgical practitioners with a comprehensive surgical repertoire, yet they lack the depth of expertise and limited access to advanced training in modern surgical techniques due to the relatively low incidence of the respective diseases. In smaller, peripheral German hospitals, it is not uncommon for general surgeons to perform a wide range of surgical procedures, which impedes the development of adequate specialization.

Third, the number of procedures performed at each institution may also have a significant impact, as a sufficient number of cases is required to complete the learning curve in a timely manner. The duration of the learning curve for laparoscopic rectal resection is reported to be approximately 50 procedures, while 32–75 procedures are required for the robotic technique [25]. Laparoscopic colon surgery with medial-to-lateral dissection requires approximately 38 procedures [26]. A low volume of procedures and the relatively long operation times at the beginning of the learning curve may be contributing factors to the slow introduction of new techniques. The relatively high density of physicians, hospitals, and beds per capita in Germany may contribute to the slow adoption of minimally invasive surgery for colorectal cancer, particularly given the associated learning curve.

Finally, the introduction of working time regulations in Germany may result in a reduction in the time spent on training procedures. This could potentially lead to a decline in the number of colorectal procedures performed during surgical training, which may, in turn, affect the learning of complex minimally invasive surgical techniques. Although limiting weekly working hours has been shown to improve patient safety, as evidenced by a reduction in treatment errors and adverse events, in some countries, the revised regulations have had a detrimental impact on the training experience of surgical residents.

Regardless of the reasons for the slow expansion of the use of minimally invasive colorectal surgery in Germany, our results also support the hypothesis that minimally invasive colorectal surgery significantly decreases the length of hospital stay by approximately six days and also reduces in-hospital mortality and morbidity. Our data are therefore consistent with the meta-analysis published in 2005 by Schwenk and colleagues [4], who were able to confirm a reduction in hospital stay of 1.5 days with the minimally invasive approach in the early phase of the introduction of laparoscopic colorectal surgery. However, it must be emphasized that the variability of the postoperative hospital stay was very high and varied in the included studies between 3.9 and 10.4 days in the laparoscopic arm and between 6 and 12.7 days in the conventional open arm. In our group, the length of hospital stay was 19.5 ± 14.8 days in the open group and 11.0 ± 9.2 days in the laparoscopic group; there was no difference according to tumor location (colon versus rectum). However, it should be noted that we analyzed the total hospital stay and not the postoperative stay.

Interestingly, we observed a significantly higher prevalence of acute post-hemorrhagic anemia in the laparotomy group, which can be considered as a consequence of increased intraoperative or postoperative blood loss. This observation is consistent with the study by Kiran and colleagues [27], who reported that patients undergoing open colon surgery experienced greater intraoperative blood loss and transfusion requirements than patients treated laparoscopically. Furthermore, we found a significantly higher prevalence of respiratory insufficiency in the group of patients who underwent conventional open surgery. Suppression of pulmonary function is a known consequence of open abdominal surgery and was first described in 1933 [28]. In this context, Milsom et al. [29] described that more than half of their laparoscopic patients achieved 80% of preoperative forced vital capacity (FVC) within three postoperative days, whereas patients in the open group required six days to achieve the same goal. Other abdominal surgical procedures, such as laparoscopic cholecystectomy, are known to have better postoperative lung function [30].

However, we were surprised by the significantly lower use of a surgical robot for colorectal surgery in the hospitals included in our database. Although a recently published RCT failed to demonstrate a difference in the quality of total mesorectal excision (TME) for RAS compared to laparoscopic rectal resection [31], the short-term results of the robotic versus laparoscopic surgery for middle and low rectal cancer (REAL) trial suggests that RAS leads to a better oncologic quality of resection compared to conventional laparoscopic surgery [32]. This includes also a macroscopically complete resection and a negative circumferential resection margin. In addition, RAS is associated with less surgical trauma and better postoperative recovery [32]. In addition, a RCT from Denmark demonstrated that patients who underwent robotic resection of the rectum required less perioperative analgesia than patients who had surgery laparoscopically [33].

It is important to note that when comparing the various surgical techniques in colorectal surgery (i.e., open, laparoscopic, and robotic), the advantages and disadvantages of each technique must be considered [34]. The advantage of open surgery is the direct visualization of a larger surgical field, which can be particularly advantageous in the case of extensive and metastatic tumors. However, the open approach is sometimes more advantageous when there are very extensive adhesions that do not allow a minimally invasive approach. In addition, direct palpation of the organs allows the surgeon to get a tactile feel for the tissue. Laparoscopic surgery uses smaller incisions, resulting in less tissue damage, less scarring, faster healing, and better cosmetic results. This results in less postoperative pain, allowing for earlier mobilization, reduced use of pain medication, and a shorter hospital stay. Laparoscopic surgery uses a camera that provides a higher morphological quality of a magnified, surgical area. This improves precision and reduces the risk of complications. RAS offers the benefits of laparoscopic surgery with those of open surgery (high-quality three-dimensional view) and can be particularly important in complex procedures, providing greater precision and control of surgical instruments. In addition, it offers a more ergonomic working environment for the surgeon, thus minimizing the risk of fatigue [35, 36].

Although our study included a large multicenter cohort of patients, it is important to acknowledge the limitations of our research. First, although the study was multicenter based on 36 hospitals, these hospitals are only a small proportion of approximately 2000 hospitals in Germany. Second, our cohort consisted of patients who were admitted to the hospital with a primary diagnosis of CRC. Consequently, we included patients who were admitted electively for colon resection as well as patients who may have required emergency surgery due to tumor-related complications (e.g., ileus, perforation, bleeding). Thus, the proportion of patients who underwent conventional open surgery may have been in a poorer general condition and with inadequate preoperative preparation, which probably led to a higher complication rate, mortality, and length of hospital stay. Furthermore, the higher proportion of patients with lymph node and distant metastases among the laparotomized cohort may indicate that more complex procedures with synchronous metastatic surgery were performed in this group. Moreover, it is unclear whether the hospitals included were certified CRC centers, how many CRC surgeries they performed annually, and whether each department had a robotic system. In addition, the database used does not contain detailed information on factors influencing surgical approach, including economic variables or information on the special training and experience of physicians in respective hospitals. Finally, our study is based on a population-based descriptive analysis, which inherently limits the ability to establish causal relationships. Consequently, the results are primarily epidemiological and observational, which constrains the depth of conclusions that can be drawn. The results presented reflect specific practices in Germany and may have limited applicability to global CRC surgery.

However, our findings suggest that future research could examine the impact of specialized training programs on the adoption of minimally invasive surgical techniques. An investigation into the potential benefits of simulation models and surgical training programs for laparoscopic and robotic surgery could provide valuable insights for enhancing surgical training and patient outcomes. Integrating comprehensive laparoscopic and robotic surgery training into the standard surgical residency curriculum could help ensure that new surgeons are proficient in these techniques. The development of standardized certification programs could also promote consistency and quality of care across institutions. The establishment of specialized departments for CRC surgery in hospitals and the concentration of complex cases in high-volume centers that also utilize modern minimally invasive surgical techniques and provide training for young surgeons could prove an effective strategy for improving surgical outcomes. A greater surgical volume facilitates more rapid attainment of proficiency and completion of the learning curve for advanced surgical techniques. Moreover, adjusting work schedules to balance training needs and patient safety can help optimize surgical outcomes. The establishment of collaborative networks between hospitals to share data and best practices can accelerate the adoption of minimally invasive techniques. These networks could also facilitate the conduct of multicenter studies that provide robust evidence for clinical practice. By addressing these areas, future research and improvements in surgical practice can lead to better patient outcomes, lower healthcare costs, and wider acceptance of minimally invasive techniques in CRC surgery.

In conclusion, despite the demonstrated benefits of minimally invasive techniques for CRC surgery, the majority of such procedures in Germany are still performed using the traditional open approach. This study highlights the significantly lower prevalence of minimally invasive surgeries, including RAS, compared to international standards. Nonetheless, the findings underscore the advantages of minimally invasive surgery, which include reduced in-hospital mortality, fewer complications, and shorter hospital stays, indicating a need for broader adoption and better training for these advanced surgical techniques.
